# The *gigA/gigB* Genes Regulate the Growth, Stress Response, and Virulence of *Acinetobacter baumannii* ATCC 17978 Strain

**DOI:** 10.3389/fmicb.2021.723949

**Published:** 2021-08-04

**Authors:** Hua Zhou, Michael J. Gebhardt, Daniel M. Czyz, Yake Yao, Howard A. Shuman

**Affiliations:** ^1^Department of Respiratory and Critical Care Medicine, The First Affiliated Hospital, Zhejiang University School of Medicine, Hangzhou, China; ^2^Department of Microbiology, University of Chicago, Chicago, IL, United States; ^3^Department of Microbiology and Cell Science, University of Florida, Gainesville, FL, United States

**Keywords:** *Acinetobacter baumannii*, nitrogen phosphotransferase system, *Galleria mellonella*, *gigA*, *gigB*, *ptsP*

## Abstract

*Acinetobacter baumannii* is an important pathogen of nosocomial infection. Recently, a group of genes, named “*gig*” (for Growth in *Galleria*), have been identified in a contemporary multi-drug resistant clinical isolate of *A. baumannii*—strain AB5075. Among these so-called *gig* genes, *gigA* and *gigB* were found to promote antibiotic resistance, stress survival, and virulence of AB5075 by interacting with the nitrogen phosphotransferase system (PTS^Ntr^). This study aimed to investigate the roles of *gigA*/*gigB*, which appear to comprise a stress-signaling pathway (encoding for an atypical two-component system response regulator and a predicted anti-anti-sigma factor, respectively), and the involvement of *ptsP* (encoding the Enzyme I component of the PTS^Ntr^) in the growth, stress resistance, and virulence of the widely studied *A. baumannii* strain ATCC 17978. Genetic analyses of strains harboring mutations of *gigA* and *gigB* were performed to investigate the roles of these genes in bacterial growth, stress resistance, evading macrophage defense, and killing of *Galleria mellonella* larva. In contrast with findings from strain AB5075 where *gigA* and *gigB* contribute to aminoglycoside resistance, the data presented herein indicate that the loss of *gigA/gigB* does not impact antibiotic resistance of strain ATCC 17978. Interestingly, however, we found that deletion of *gigA/gigB* in the ATCC 17978 background imparts a general growth in laboratory medium and also conferred growth and replication defects within murine macrophages and an inability to kill *G. mellonella* larvae. Importantly, studies as well as the loss of *ptsP* restored the phenotypes of the *gigA/gigB* mutant to that of the wild-type. The data presented herein indicate that in *A. baumannii* ATCC 17978, the *gigA/gigB* genes play a key role in both growth and virulence traits, but are dispensable for other stress-resistance survival phenotypes, including aminoglycoside resistance. Our findings thus highlight several similarities and also important differences between the *gigA/gigB* stress-signaling pathway in two commonly studied isolates of this troublesome pathogen.

## Introduction

*Acinetobacter baumannii* is a Gram-negative bacterium responsible for approximately 20% of intensive care unit infections worldwide and is the top-ranking pathogen on the World Health Organization’s list of priority antibiotic-resistant pathogens (Lee et al., 2017; WHO, 2017; Karalewitz and Miller, 2018). Many circulating *A. baumannii* strains exhibit a multidrug-resistant phenotype due to a combination of intrinsic and acquired traits (Peleg et al., 2012; Gottig et al., 2014). Identification of virulence determinants and understanding of the mechanisms underlying the pathogenesis of *A. baumannii* are important for combating *A. baumannii* infection.

Recently, Gebhardt et al. (2015) have identified a group of genes, named “*gig*” (for Growth in *Galleria*), that are required for growth of the highly virulent and highly antibiotic resistant *A. baumannii* strain AB5075 in *Galleria mellonella* larvae. Among these genes, *gigA* and *gigB* were found to promote antibiotic resistance, stress survival, and virulence of AB5075 by interacting with the nitrogen phosphotransferase system (PTS^Ntr^) (Gebhardt and Shuman, 2017). *gigA* encodes a protein phosphatase 2C-type phosphatase, and *gigB* encodes a putative anti-anti-sigma factor. GigA was shown to dephosphorylate GigB, which in turn regulates the phosphate level on NPr, a key component of the PTS^Ntr^. Disruption of the GigA/GigB signaling pathway led to the altered expression of numerous stress response genes. Thus, the intersection of GigA/GigB with the PTS^Ntr^ promotes stress survival (Gebhardt and Shuman, 2017).

The *ptsP* gene encodes the enzyme I component of the PTS^Ntr^. Mutations in *ptsP* increases tobramycin resistance (Schurek et al., 2008; Scribner et al., 2020; Abisado et al., 2021). In AB5075, deletion of *ptsP* in either a Δ*gigA* or Δ*gigB* background suppresses the *gig* mutant phenotypes to near-wild-type levels, including restoration of aminoglycoside resistance, stress survival, and growth in *Galleria* larvae (Gebhardt and Shuman, 2017). Our previous work has revealed that in *A. baumannii* AB5075 mutants lacking both *gigA* and *gigB* (i.e., a Δ*gigAB* double mutant), only concurrent complementation of both *gigA* and *gigB* can restore kanamycin resistance to wild-type levels, suggesting that *gigA* and *gigB* are inseparable in the pathogenesis of *A. baumannii* (Gebhardt and Shuman, 2017). However, the role played by *ptsP* in the survival and virulence of an *A. baumannii* Δ*gigAB* mutant strain remains unknown.

ATCC 17978 is among the best-studied strains of *A. baumannii* and is an ideal model for genetic manipulation compared with clinical isolates due to its sensitivity to most antibiotics and high genome homology to current *A. baumannii* isolates (Sahl et al., 2011; Jacobs et al., 2014a). In this study, we investigated the roles of *gigA*/*gigB* and the involvement of *ptsP* in the growth, stress response, and virulence of ATCC 17978. Our results may provide new information about the roles of *gigA*/*gigB* and the PTS^Ntr^ system in the pathogenesis of *A. baumannii* infection.

## Materials and Methods

### Bacterial Strains and Culture

*A. baumannii* ATCC 17978 was purchased from The American Type Culture Collection (Manassas, VA, United States). *Escherichia coli* DH5α was obtained from Invitrogen (Carlsbad, CA, United States). The tetracycline-resistant and sucrose-sensitive plasmid pMJG42, apramycin-resistant pMJG120, and gentamicin-resistant pMJG125 plasmids were kept in our laboratory at the University of Chicago (Chicago, IL, United States). The bacteria were cultured in lysogeny broth (LB) medium at 37°C. When required, the antibiotics added for selection were tetracycline (10 μg/mL), apramycin (50 μg/mL), and gentamicin (10 μg/mL).

### Generation of Gene Deletion and Complementation Plasmids

Gene deletion and complementation plasmids were generated as previously described (Jacobs et al., 2014b; Gebhardt et al., 2015; Gebhardt and Shuman, 2017). Briefly, gene deletions were performed using allelic exchange plasmid pMJG42. The resulting plasmids (pMJG42-Δ*gigAB*, pMJG42-Δ*ptsP*) were transformed into ATCC 17978 via electroporation to obtain ATCC 17978 Δ*gigAB* and ATCC 17978 Δ*ptsP* mutants. After tetracycline selection and sucrose counterselection, the clones were subjected to colony PCR. Gene deletions were confirmed by sequencing. For complementation of the deleted *gigA*/*gigB*, the entire open reading frames of *gigA*/*gigB* were amplified by PCR, cloned into pMJG120 or pMJG125 to obtain pMJG120-*gigAB* or pMJG125-*gigAB*, and transformed into ATCC 17978 Δ*gigAB* via electroporation to obtain ATCC 17978 Δ*gigAB* pMJG120-*gigAB* or ATCC 17978 Δ*gigAB* pMJG125-*gigAB.*

ATCC 17978 Δ*gigAB* was transformed with pMJG42-*gigA*/*gigB* to generate ATCC 17978’ with *in situ* complementation of *gigA* and *gigB*. ATCC 17978 Δ*ptsP* was transformed with *pMJG42-gigAB* to generate ATCC 17978 Δ*ptsP*Δ*gigAB.* All bacterial strains, plasmids, and primers in this study were summarized in Supplementary Tables 1–3.

### Whole-Genome Sequencing

Eight strains of ATCC 17978 Δ*gigAB* were randomly selected from different batches for whole-genome sequencing. Genomic DNA was prepared using the QIAamp DNA Mini Kit (Qiagen, Germany) and then subjected to whole genome sequencing (WGS) using the Illumina Hiseq2500 platform (Illumina, CA, United States) following the 2 × 100 bp protocol. The average sequencing throughput was 1 Gb. Raw fastq reads were trimmed by Trimmomatic for quality control (Bolger et al., 2014) and subsequently mapped against the reference genome of ATCC 17978-mff (Accession No. CP012004) with Bowtie2 (Langmead and Salzberg, 2012). Variant calling was performed using the bcftools call function with the default parameters (Danecek et al., 2021). We had submitted all of these data to NCBI BioProject database under the BioProject ID PRJNA738724.^1^

### Calculation of Gene Deletion Efficiency

After antibiotics selection and sucrose counterselection, 24 clones were randomly selected for colony PCR. Gene deletions were confirmed by sequencing. The gene deletion efficiency was calculated as (the number of the clones with successful deletion mutation)/24 × 100% The experiment was repeated three times, and data were expressed as the mean ± standard deviation (SD).

### Efficiency of Plating Analysis

Overnight cultures of the indicated strains were back-diluted into fresh LB and grown for 2 h. After outgrowth, aliquots of the cultures were serially diluted. Then, a 10-μL aliquot was spotted onto LB agar plates with or without stressors as follows: HCl (medium adjusted to pH 5.5), ZnCl_2_ (final concentration = 1.25 mmol/L). Colony forming units (CFU) were counted at 12 h after incubation at 37 or 50°C. Efficiency of plating (EOP) was calculated as (CFU recovered on stress medium)/(CFU recovered on plain medium at 37°C).

### Bacterial Growth Curves

ATCC 17978, ATCC 17978 Δ*gigAB*, ATCC 17978 Δ*ptsP*, and ATCC 17978 Δ*ptsP* Δ*gigAB* were cultured in LB medium without antibiotics. ATCC 17978 Δ*gigAB* pMJG120 and ATCC 17978 Δ*gigAB* pMJG120-*gigAB* were cultured in LB medium containing 50 mg/L apramycin. ATCC 17978 Δ*gigAB* pMJG125 and ATCC 17978 Δ*gigAB* pMJG125-*gigAB* was cultured in LB medium containing 10 mg/L gentamicin, in the presence or absence of 1% (w/v) arabinose.

Each strain was grown overnight on the appropriate LB agar plate, and a single colony was picked and expanded in 2 mL LB broth overnight. A 1 μL aliquot was diluted at 1:1,000, and the dilution was added into triplicate wells of a 96-well plate at 200 μL/well. LB medium without bacteria was used as a blank. The OD_600_ was determined every 15 min using a Biotek plate reader (Winooski, VT, United States). Growth curves were generated using GraphPad Prism 5 (San Diego, CA, United States). Each experiment was performed in triplicate and repeated three times. The mean was calculated for each experiment, and data were presented as the mean of three experiments.

### Antibiotic Sensitivity Testing

Antibiotic sensitivity testing was performed as previously described (Gebhardt et al., 2015). The antibiotics used in this study are summarized in Table 1. Data were expressed as minimum inhibitory concentration (MIC).

**TABLE 1:** Antibiotic susceptibilities of deletion strains (MIC, mg/L).

	**ATCC 17978**	**ATCC 17978**Δ***ptsP***	**ATCC 17978**Δ***ptsP***Δ***gigAB***	**ATCC 17978 **Δ***gigAB***
Ampicillin	>128	128	>128	>128
Apramycin	8	4	4	2
Chloramphenicol	64	64	32	32
Gentamicin	0.5	0.5	0.5	< 0.25
Hygromycin	128	128	128	128
Kanamycin	2	2	1	1
Streptomycin	16	16	16	16
Tetracycline	0.5	1	0.5	0.5
Polymyxin B	0.5	1	0.5	0.5

*MIC, minimum inhibitory concentration.*

### Isolation of Mouse Bone Marrow-Derived Macrophages (BMDMs)

Bone marrow-derived macrophages (BMDMs) were obtained from 8 to 12 week old female C57BL/6J (Jackson Laboratories) mice as previously described (Toda et al., 2021). Briefly, bone marrow cells were collected from the femur and tibia of mice and maintained in RPMI 1640 medium (Gibco, Thermo Fisher Scientific, Waltham, MA, United States) supplemented with 10 ng/mL mouse macrophage colony-stimulating factor (mMCSF; Gibco), 10% fetal bovine serum (Gibco), and 1% Pen/Strep (Gibco) at 37°C in a humidified atmosphere of 5% CO_2_ for 7 days.

### Bacterial Killing Assay

Mouse BMDMs were plated in a 96-well plate at a density of 50,000 cells/well and cultured overnight. Cells were infected with wild-type ATCC 17978, ATCC 17978 Δ*gigAB*, or ATCC 17978 Δ*gigAB* pMJG120-*gigAB* at 5 × 10^5^ CFU/mL. The plate was centrifuged at 2,170 rpm for 30 min at room temperature, followed by incubation at 37°C for 30 min. After replacing the medium with RPMI 1640 containing 100 mg/L gentamicin to kill extracellular bacteria, the infected cells were incubated for an additional 1 h (*t* = 0 h). Then, the infected cells were cultured in RPMI 1640 supplemented with 25 mg/L gentamicin (wild type and Δ*gigAB* strains) or 1.5 mg/L polymyxin (Δ*gigAB* pMJG120-*gigAB* strain). Cell lysates were collected at 0, 2, and 6 h post infection using phosphate buffered saline (PBS) containing 1% Triton-X100, serially diluted, and plated on LB agar plates. CFU were enumerated after 18 h of growth at 37°C. Each experiment was performed in triplicate and repeated three times. The mean CFU of surviving bacteria was calculated for each experiment, and data were presented as the mean of three experiments.

### *G. mellonella* Killing Assay

Infection of *Galleria mellonella* larvae (Knutson’s LiveBait, Brooklyn, MI) was performed as described previously (Jacobs et al., 2014a; Gebhardt and Shuman, 2017). Briefly, the bacteria were grown overnight in an orbital shaker (37°C, 200 rpm), and overnight cultures were resuspended in PBS to a final OD_600_ of 1.0. *G. mellonella* larvae were randomly divided into three groups (*n* = 10/group). A total of 10 μL cultures (5 × 10^6^ CFU/mL) were inoculated into the last left proleg of each larva. After injection, larvae were incubated at 37°C. The number of dead larvae was recorded hourly. Each experiment was performed in triplicate and repeated three times. The mean larval survival was calculated for each experiment, and data were presented as the mean of three experiments.

### Statistical Analysis

Data were expressed as the mean ± SD. Statistical analysis was performed using GraphPad Prism 5. Differences among groups were compared using one-way ANOVA followed by Dunnett’s *post-hoc* test. Killing curves were plotted using the Kaplan-Meier method. A *P*-value of < 0.05 was considered statistically significant.

## Results

### *gigA/gigB* Are Important for the Growth but Not Required for the Survival of ATCC 17978

In our preliminary study, we noticed that the *gigA/gigB* deletion efficiency in wild-type 17978 was only 4.2%, suggesting that loss of *gigA/gigB* inhibits the growth of 17978 (Table 2). To further explore how the genetic background affects the efficiency of *gigA/gigB* deletion, we assessed Δ*gigAB* mutation efficiency in various 17978 genetic backgrounds. All gene deletions and complementation were confirmed by sequencing. The results of these analyses are shown in Table 2, and indicate that strains harboring either a *ptsP* deletion or *in trans-*complementation of *gigA/gigB* greatly increased the frequency of isolating the *gigA/gigB* double deletion mutation, suggesting that *ptsP* deletion and *gigA/gigB* complementation can compensate for the apparent growth defect caused by loss of *gigA/gigB.*

**TABLE 2:** The efficiency of *gigA/gigB* deletion in ATCC 17978 of different gene background.

**Gene background**	**Extrachromosomal *gigA/gigB* expression**	***gigA/gigB* deletion efficiency (%)**
ATCC17978	−	4.2
ATCCATCC17978*ΔptsP*	−	52.1
ATCC17978 pMJG120	−	4.2
ATCC17978 pMJG120-*gigAB*	+	47.8
ATCC17978*ΔptsP* pMJG120	−	68.8
ATCC17978*ΔptsP* pMJG120-*gigAB*	+	42.9
ATCC17978’	−	8.4
ATCC17978 pMJG125-*gigAB* (with 1% arabinose)	+	54.2
ATCC17978 pMJG125-*gigAB* (without 1% arabinose)	+/−	Large colonies: wild-type Small colonies: *ΔgigAB*

In the arabinose-inducible pMJG125 vector-based complementation of *gigA/gigB* background, we observed that the Δ*gigAB* colonies were smaller than wild-type colonies in the absence of arabinose; this phenotype was eliminated by the supplementation with 1% arabinose (Table 2 and Supplementary Figure 1). This finding further confirms that *gigA/gigB* are important for the growth of 17978 and that complementation of *gigA/gigB* with arabinose supplementation promotes the growth of Δ*gigAB* mutant to the wild-type level.

To determine if the loss of *gigA/gigB* required the generation of suppressing mutations, we performed whole genome sequencing on eight 17978 Δg*igAB* clones isolated from different batches of gene knockout experiments. We found that, other than the *gigA/gigB* deletion, the genome of each of the sequenced Δg*igAB* clones was 100% identical to the genome of ATCC 17978-mff reference strain, suggesting that deletion of the *gigA/gigB* genes does not require suppressing/compensatory mutations and that ATCC 17978 can survive without *gigA/gigB.* Thus, *gigA/gigB* are important for the growth but not required for the survival of ATCC 17978 under our routine laboratory culturing conditions.

### Loss of *ptsP* and/or *gigAB* Does Not Affect Antibiotic Resistance of ATCC 17978

To explore the roles of *ptsP* and *gigA/gigB* in the antibiotic resistance of ATCC 17978, we performed antibiotic susceptibility tests in the wild type and gene deletion strains. As shown in Table 1, although the MIC of apramycin, chloramphenicol, and kanamycin were decreased in at least two deletion mutation strains compared with those in wild-type ATCC 17978, the results did not reach statistical significance. These data suggest that, in contrast to our previous findings in the *A. baumannii* AB5075 strain background, *ptsP* and *gigA/gigB* are not required for antibiotic resistance of ATCC 17978.

### Loss of *ptsP* Restores the Growth of 17978 Δ*gigAB* to the Wild-Type Level

**FIGURE 1:**
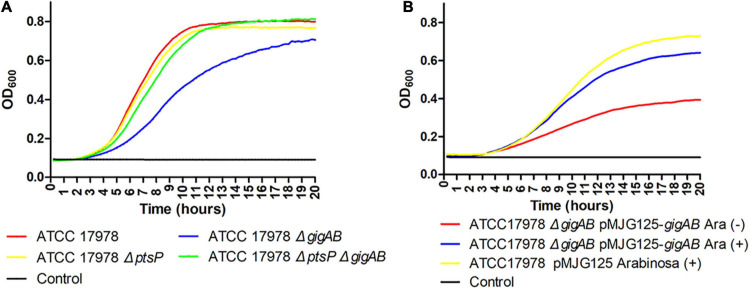
Growth curves of different ATCC 17978 stains. **(A)** The indicated strains were grown for 20 h at 37°C in LB. **(B)** The strains indicated were grown for 96 h at 37°C in LB with or without 1% arabinose. Growth was measured by determining the OD_600_ every 15 min. Each experiment was performed in triplicate and repeated three times, and the most representative curves were presented.

To explore the involvement of *ptsP* in *gigA/gigB***-**mediated growth of ATCC 17978, we performed growth curve analyses. As shown in Figure 1A, 17978 Δ*gigAB* exhibited remarkably suppressed growth compared with the wild-type, whereas 17978 Δ*ptsP* exhibited a comparable growth rate to the wild-type, suggesting that *gigA/gigB* contribute to 17978 growth. Interestingly, 17978 Δ*ptsP*Δ*gigAB* showed comparable growth to the wild-type strain, indicating that loss of *ptsP* alleviates the growth defect associated with the loss of *gigA/gigB*. In addition, pMJG125-based complementation of *gigA/gigB* also restored the growth of 17978 Δ*gigAB* to the wild-type level in the presence of arabinose (Figure 1B).

### *gigA*/g*igB* Mediate *in vitro* High-Temperature Resistance of ATCC 17978

**FIGURE 2:**
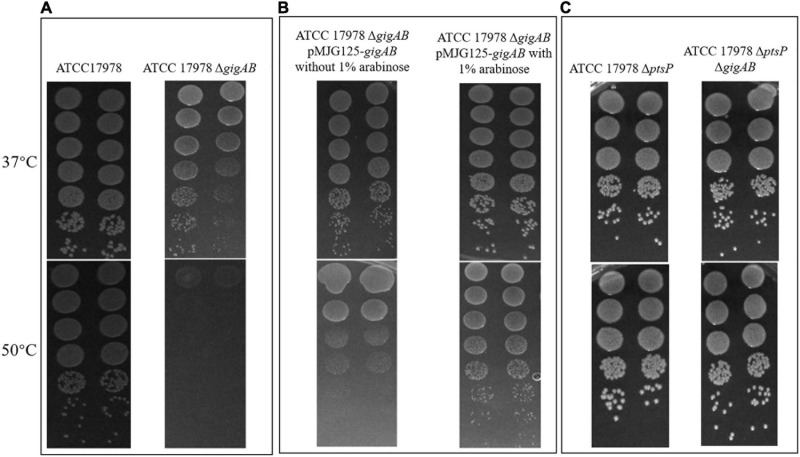
The roles of *gigA/gigB* in ATCC 17978 in response to high temperature. **(A)** Images of wild-type ATCC 17978 and ATCC 17978 Δ*gigAB* mutant grown on LB at 37 or 50°C. **(B)** Images of ATCC 17978 Δ*gigAB* pMJG125-*gigAB* grown on LB without or with 1% arabinose at 37 or 50°C. **(C)** Images of ATCC 17978 Δ*ptsP* and ATCC 17978 Δ*ptsP*Δ*gigAB* grown on LB at 37 or 50°C.

We next sought to explore any additional roles of *gigA*/g*igB* and *ptsP* in stress resistance of ATCC 17978. Although the wild-type 17978 grown at 50°C showed a moderately reduced colony size phenotype when compared with those grown at 37°C, no significant loss of CFU was observed (EOP = 1; Figure 2A, left panel). However, loss of both *gigA* and *gigB* resulted in a dramatic reduction in CFU at 50°C (EOP = 10^–5^; Figure 2A, right panel), suggesting that *gigA* and *gigB* contribute to high-temperature resistance of 17978. In the absence of arabinose, complementation of both *gigA* and g*igB* partially restored the growth of Δ*gigAB* mutant at 50°C (EOP = 10^–3^; Figure 2B, left panel). Importantly, arabinose supplementation further restored the growth of Δ*gigAB* mutant with *gigA/gigB* complementation to the wild-type level at 50 °C (EOP = 1; Figure 2B, right panel), despite the small sizes of the colonies. This finding suggests that *gigA/gigB* contribute to high-temperature resistance of 17978 on solid media. As observed in the growth curves described above, 17978 Δ*ptsP* Δ*gigAB* and 17978 Δ*ptsP* exhibited comparable growth at 50°C (EOP = 1; Figure 2C), suggesting that loss of *ptsP* restores the growth of Δ*gigAB* mutant under high-temperature stress. Taken together, these results suggest that *gigA/gigB* mediate high-temperature resistance of 17978 on LB agar plates, whereas *ptsP* negatively regulates this response.

**FIGURE 3:**
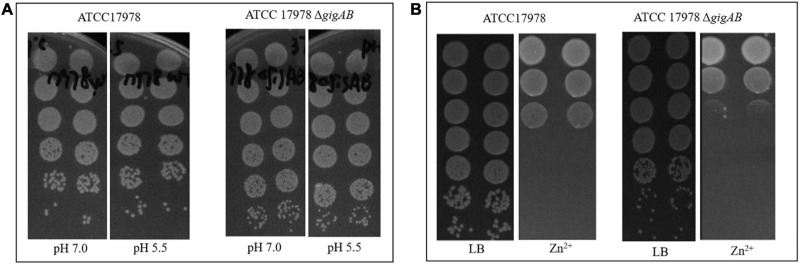
The roles of *gigA/gigB* in ATCC 17978 in response to acid or zinc. **(A)** Images of wild-type ATCC 17978 and ATCC 17978 Δ*gigAB* mutant grown on LB at pH 7.0 or pH 5.5. **(B)** Images of wild-type ATCC 17978 and ATCC 17978 Δ*gigAB* mutant grown on LB with or without 1.25 mmol/L Zn^2+^.

When we examined the ability of the Δ*gigAB* strain to survive acid stress (pH = 5.5), we did not observe significant differences in the growth between the wild-type and Δ*gigAB* mutant strains (EOP = 1; Figure 3A), suggesting that the 17978 strain is not sensitive to pH stress as measured herein, and that the loss of *gigA* and *gigB* does not confer an acid stress sensitivity on the 17978 strain.

When cultured on LB containing Zn^2+^, both wild-type and Δ*gigAB* mutant demonstrated significantly suppressed growth compared with those cultured on LB without Zn^2+^ (EOP = 10^–4^, Figure 3B). No major difference was observed in the growth between the wild-type and Δ*gigAB* mutant. This finding suggests that factors other than *gigA* and *gigB* mediate zinc resistance of ATCC 17978.

### *gigA/gigB* Protect ATCC 17978 From BMDM Killing

**FIGURE 4:**
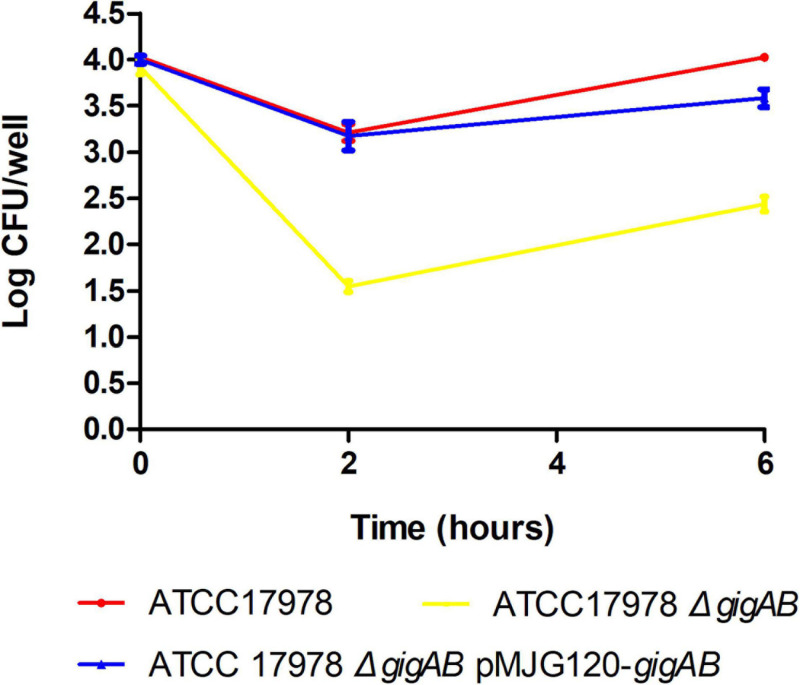
Bone marrow-derived macrophage killing of wild-type and mutant ATCC 17978. Murine bone marrow derived macrophage (BMDMs) were cultured for 24 h, then challenged with wild-type ATCC 17978, ATCC 17978 Δ*gigAB*, or ATCC 17978 Δ*gigAB* pMJG120*-gigAB* at 5 × 10^4^ colony forming unit count (CFU)/mL in the presence of 1 mM IPTG. BMDMs were lysed at 0, 2, or 6 h after challenge and surviving bacteria were quantified via standard plate count method.

Evading macrophage phagocytosis is critical for the survival of pathogens *in vivo* (Rosales and Uribe-Querol, 2017). To investigate the roles of *gigA/gigB* in macrophage killing evasion of ATCC 17978, we infected murine BMDMs with the wild-type, *ΔgigAB* mutant, and *gigAB* complementation strains and monitored their survival and replication. When BMDMs were infected with wild-type 17978, we observed a 10-fold reduction of intracellular live bacteria at 2 h after infection (Figure 4). On the other hand, when BMDMs were infected with *ΔgigAB* mutant, we observed a 300-fold reduction of live bacteria in BMDMs at 2 h after infection (Figure 4), suggesting a decreased replication ability of the *ΔgigAB* mutant. Importantly, the Δ*gigAB* pMJG120-*gigAB* complementation strain exhibited a similar trend of survival and replication and comparable CFU at different time points to the wild-type 17978 (Figure 4). These results suggest that *gigA* and *gigB* promote macrophage killing evasion of ATCC 17978.

### *gigA/gigB* Are Required for Killing *G. mellonella*

**FIGURE 5:**
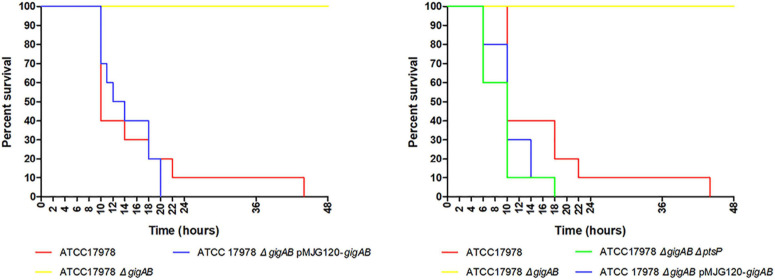
Killing of *G. mellonella* larvae. *G. mellonella* larvae were inoculated with 5 × 10^6^ CFU/mL of wild-type ATCC 17978, ATCC 17978 Δ*gigAB*, or ATCC 17978 Δ*gigAB* pMJG120*-gigAB* (*n* = 10 larvae/group). After injection, larvae were incubated at 37°C. The number of dead larvae was recorded hourly.

To examine the roles of *gigA/gigB* in the virulence of ATCC 17978, we performed a *G. mellonella* killing assay. As shown in Figure 5, inoculation of *G. mellonella* larvae with wild-type 17978 resulted in a rapid killing of the larvae starting 8 h after inoculation. No killing was observed in the larvae that received Δ*gigAB* mutant within 48 h after inoculation. Complementation of both *gigA* and *gigB* restored the virulence of bacteria to nearly wild-type level. Thus, *gigA/gigB* are required for the virulence of ATCC 17978. Much like for the growth and temperature studies described above, both the 17978 *ΔpstP* and 17978 *ΔptsP ΔgigAB* strains killed larvae with similar kinetics as the wild-type 17978 strain, suggesting that the loss of *ptsP* restores the virulence defect caused by the Δ*gigAB* deletion.

## Discussion

In this work, we sought to investigate the roles of *gigA*/*gigB* in the survival, stress resistance, macrophage killing evasion, and virulence of *A. baumannii* ATCC 17978 as well as the involvement of *ptsP* in *gigA*/*gigB* signaling. We found that *gigA*/*gigB* are important for growth of *A. baumannii* ATCC 17978, but are not explicitly required for survival of 17978. Indeed, the Δ*gigAB* mutant strain exhibited growth defects at both 37°C and 50°C compared with the wild-type strain, which was effectively restored by pMJG125-based *gigA/gigB* complementation in the presence of arabinose or loss of *ptsP.* Furthermore, *gigA*/*gigB* protected 17978 from murine BMDM killing and were required for the virulence of 17978 in *G. mellonella.*

Bacterial genetics remains an important and powerful tool for revealing the function(s) of specific genes. Efficient construction of gene knockouts or other types of mutations in bacteria often requires modifications of genetic background (Xu and Zhang, 2016). Our preliminary data have shown that the *gigA/gigB* deletion efficiency in wild-type 17978 was only 4.2%, suggesting that *gigA/gigB* are critical for the survival of ATCC 17978. We further found that *gigA/gigB* complementation or *ptsP* deletion significantly improved *gigA/gigB* deletion efficiency, suggesting that *gigA/B* complementation or *ptsP* deletion compensates for the lack of *gigA/B* in 17978. This is consistent with our previous study showing that loss of *ptsP* in the *A. baumannii*Δ*gigA* or Δ*gigB* mutant restores the growth of *A. baumannii* in *G. mellonella* larvae (Gebhardt and Shuman, 2017).

When studying genes essential for bacterial growth and/or survival, it is not uncommon to inadvertently isolate clones which harbor compensatory or suppressing mutations that alleviate the phenotype of the particular genes being studied (MacLean and Vogwill, 2014). To exclude the possibility that the Δ*gigAB* strain acquired such compensatory mutations, we performed whole genome sequencing in multiple independently derived Δ*gigAB* clones and found that, with the exception of the *gigA/gigB* deletion, the genome of Δ*gigAB* clones displayed 100% identity to the genome of the wild-type strain, suggesting that *gigA/gigB* deletion does not require subsequent compensatory mutations, further confirming that 17978 can survive without *gigA/gigB*.

When we knocked out *gigA/gigB* in the genetic background of pMJG125-*gigAB* conditional strain, we observed that in the absence of arabinose, the colonies of Δ*gigAB* mutants were smaller than those of the wild-type (Table 2 and Supplementary Figure 1). In addition, Δ*gigAB* mutant still showed growth defect even after the complementation of *gigA/gigB* in the absence of arabinose (Figure 1). Of note, arabinose supplementation effectively reversed these effects. We attribute these observations to leaky basal expression from the arabinose-promoter on the multi-copy pMJG125 plasmid.

In addition to the growth in LB medium, we also investigated the roles of *gigA/gigB* in ATCC 17978 in response to several environmental stresses, including antibiotics, high temperature, Zn^2+^, and acid. Neither the wild-type nor the Δ*gigAB* mutant showed significant growth defect to acid stress (Figure 3A). Additionally, we did not observe significant differences for MIC values for various antibiotics (Table 1) and colony formation in the presence of Zn^2+^ (Figure 3B) between the wild-type and the mutant strain lacking both *gigA* and *gigB*. These results suggest that factors other than *gigA/gigB* regulate the responses of ATCC 17978 to antibiotics and Zn^2+^ stresses, in contrast to what was previously observed in the more virulent AB5075 strain (Gebhardt et al., 2015; Gebhardt and Shuman, 2017; Blaschke et al., 2018). For example, it has previously been reported that the chromosomally-encoded efflux pump CraA, AdeAB efflux system, and incubation temperature regulate antibiotic resistance of ATCC 17978 (Adams et al., 2018; De Silva et al., 2018; Kroger et al., 2018). Additionally, transcriptional analyses have shown that zinc resistance efflux pumps are responsible for zinc stress response in ATCC 17978, including two cation diffusion facilitator transporters, one heavy metal efflux transporter, and one P-type ATPase (Hassan et al., 2017). That there are differential consequences of *gigA/gigB* deletion in the AB5075 background (i.e., aminoglycoside and zinc sensitivity) and the ATCC 17978 background (i.e., growth defect under routine culture conditions) suggests that some of the inputs and/or outputs of the GigA/GigB signaling pathway have diverged since the two strains separated; yet, other facets of the pathway, such as growth at elevated temperature and virulence, have remained intact. Further research will be required to understand the molecular mechanisms that underlie the different stress responses that are regulated by GigA/GigB amongst these two isolates.

Of note, our results showed that complementation of the Δ*gigAB* deletion strain with a plasmid-borne copy of *gigA/gigB* restored growth on agar plates at high temperature and that a subsequent deletion of *ptsP* in the Δ*gigAB* background also alleviated the high temperature growth defect caused by the loss of both *gigA* and *gigB* (Figure 2), consistent with previous observations in the AB5075 strain (Gebhardt and Shuman, 2017).

We finally examined the roles of *gigA/gigB* in evading macrophage phagocytosis and killing *G. mellonella* larvae. Our data indicate that *gigA/gigB* are required for 17978 in killing *G. mellonella*: no larvae died within 48 h after inoculation with Δ*gigAB* mutant. Additionally, we found that *gigA/gigB* contribute to the macrophage killing evasion of ATCC 17978, as evidenced by the decreased intracellular live bacteria and the suppressed bacterial replication in murine BMDMs infected with Δ*gigAB* mutant compared with those infected with the wild-type strain (Figure 5). As it has been reported that RNA chaperone Hfq and superoxide dismutase of ATCC 17978 also play important roles in evading macrophage phagocytosis (Heindorf et al., 2014; Kuo et al., 2017), it will be interesting to examine if the loss of *gigA* and/or *gigB* leads to altered expression of these virulence factors.

## Conclusion

In this study, we demonstrate that *gigA/gigB* are important for the growth of *A. baumannii* strain ATCC 17978, although they are not explicitly required. The *ΔgigAB* mutant exhibits growth defects at both 37 and 50°C, which can be restored either through *gigA/gigB* complementation or by loss of *ptsP*. In contrast to findings in the *A. baumannii* AB5075 background (Gebhardt and Shuman, 2017), *gigA/gigB* do not appear to alter the response of strain 17978 to antibiotics or Zn^2+^ stress. Finally, like strain AB5075, the *gigA/gigB* genes are required for the virulence traits of strain ATCC 17978 in both resisting killing by macrophage and the *G. mellonella* infection model.

## Data Availability Statement

The datasets presented in this study can be found in online repositories. The names of the repository/repositories and accession number(s) can be found below: http://www.ncbi.nlm.nih.gov/bioproject/, PRJNA738724.

## Ethics Statement

The animal study was reviewed and approved by the Ethics Committee of the University of Chicago Medical Center.

## Author Contributions

HZ performed the all stepss of experiment, analyzed the experimental data, and drafted the manuscript. MG helped construct *A. baumannii* ATCC17978 mutants. DC helped perform the BMDM isolation and bacterial killing experiments. YY analyzed whole genome sequences and helped to analyze data. MG and DC revised the manuscript. HS designed the study and revised the manuscript. All authors read and approved the final manuscript.

## Conflict of Interest

The authors declare that the research was conducted in the absence of any commercial or financial relationships that could be construed as a potential conflict of interest.

## Publisher’s Note

All claims expressed in this article are solely those of the authors and do not necessarily represent those of their affiliated organizations, or those of the publisher, the editors and the reviewers. Any product that may be evaluated in this article, or claim that may be made by its manufacturer, is not guaranteed or endorsed by the publisher.

## Footnotes

1
http://www.ncbi.nlm.nih.gov/bioproject/738724
