# Supplementary material for: The gigA/gigB Genes Regulate the Growth, Stress Response, and Virulence of Acinetobacter baumannii ATCC 17978 Strain
Source: Front Microbiol. 2021 Aug 4;12:723949. doi: 10.3389/fmicb.2021.723949 (PMC8371402; doi:10.3389/fmicb.2021.723949)
Supplement: Supplementary file 1 [file Table_1.docx]

**Supplementary Table 1. Bacterial strains**

| Strain | Description | Source or reference |
| --- | --- | --- |
| ATCC 17978 | Wild-type | Laboratory stock |
| AB5075 | *A. baumannii*, MDR, highly virulent | Gebhardt et al., 2015 |
| DH5α | *E. coli* | Laboratory stock |
| ATCC 17978 *ΔgigAB* | *A. baumannii* ATCC 17978 lacking both *gigA* and *gigB* | This work |
| ATCC 17978 *ΔptsP* | *A. baumannii* ATCC 17978 lacking *ptsP* | This work |
| ATCC 17978 *ΔptsP* *ΔgigAB* | *A. baumannii* ATCC 17978 lacking *gigA, gigB, and ptsP* | This work |
| ATCC 17978’ | *A. baumannii* ATCC 17978 lacking both of *gigA* and *gigB* with *in situ* complemented *gigA/gigB* | This work |

**Supplementary Table 2. Plasmids**

| Plasmids | Description | Source or reference |
| --- | --- | --- |
| pMJG42 | Allele exchange vector; Tet^r^ Suc^s^ | Gebhardt et al., 2015 |
| pMJG42-*ΔgigAB* | pMJG42 for *gigA/gigB* deletion | This work |
| pMJG42-*ΔptsP* | pMJG42 for *ptsP* deletion | This work |
| pMJG120 | Incompatibility group Q vector; Apra^r^ | Gebhardt et al., 2015 |
| pMJG120-*gigAB* | pMJG120 harboring *gigA* and *gigB* coding regions | This work |
| pMJG125 | Ababinose-inducible vector; Gm^r^ | Gebhardt et al., 2015 |
| pMJG125-*gigAB* | pMJG125 harboring *gigA* and *gigB* coding regions | This work |

Tet^r^, tetracycline resistance; Suc^s^, sucrose sensitivity; Apra^r^, apramycin resistance; Gm^r^, Gentamicin resistance.

**Supplementary Table 3. Primers**

| Primers | Purpose and sequence |
| --- | --- |
| MJG863 | Forward, *gigA or gigA/gigB* deletion, 5’-flanking, with *Not1* RS  CTGCATATTGAATATAGCCTGGCTA |
| MJG864 | Reverse, *gigA/gigB* deletion, 5’-flanking  TTGAAAAAGGACTACCATATCTTCT |
| *gigA* P2 | Reverse, *gigA* deletion, 5’-flanking  CAAGATCACTTTATCCATCATAGAGGATG |
| *gigA* P3 | Forward, *gigA* deletion, 3’-flanking  TTTAAGAATCAATTAATTACATAATATACGACTTA |
| *gigA* P4 | Reverse, *gigA* deletion, 3’-flanking  ATGTACAAGTTGGCGATGCT |
| *gigB* P1 | Forward, *gigB* deletion, 5’-flanking, with *Not1* RS  CATCCTCTATGATGGATAAAGTGATCTTG |
| *gigB* P2 | Reverse, *gigB* deletion, 5’-flanking  ATAGTGCCATCTCAGACTCAAGAGTCCTTA |
| *gigB* P3 | Forward, *gigB* deletion, 3’-flanking, with *Not1* RS  ACTTTATTGCTAATCTGGATGTGTATATGA |
| MJG865 | Forward, *gigA and gigB* deletion, 3’-flanking  ACTTTATTGCTAATCTGGATGTGTATATGA |
| MJG866 | Reverse, *gigA/gigB* or *gigB* deletion, 3’-flanking, with *Not1* RS  GCTGTCTGGCACCTTCTG |
| MJG867 | *gigA/gigB* 5’-1087bp  CGCTTCTGTATTAAAATATATCTCTACCCA |
| MJG868 | *gigA/gigB* 5’ +1102bp  TCAGCCTGCCGATGTTTCT |
| MJG374 | Forward, pMJG42 *Not*I RS 5’-flank  CCCGCGCGATTTACTTTTCGACC |
| MJG375 | Reverse，pMJG42 *Not*I RS 3’-flank  CTCCGCCCCGTTCGTAAGCC |

RS, restriction site.
