# Supplementary material for: The gigA/gigB Genes Regulate the Growth, Stress Response, and Virulence of Acinetobacter baumannii ATCC 17978 Strain
Source: Front Microbiol. 2021 Aug 4;12:723949. doi: 10.3389/fmicb.2021.723949 (PMC8371402; doi:10.3389/fmicb.2021.723949)
Supplement: Supplementary file 2 [file Table_2.docx]

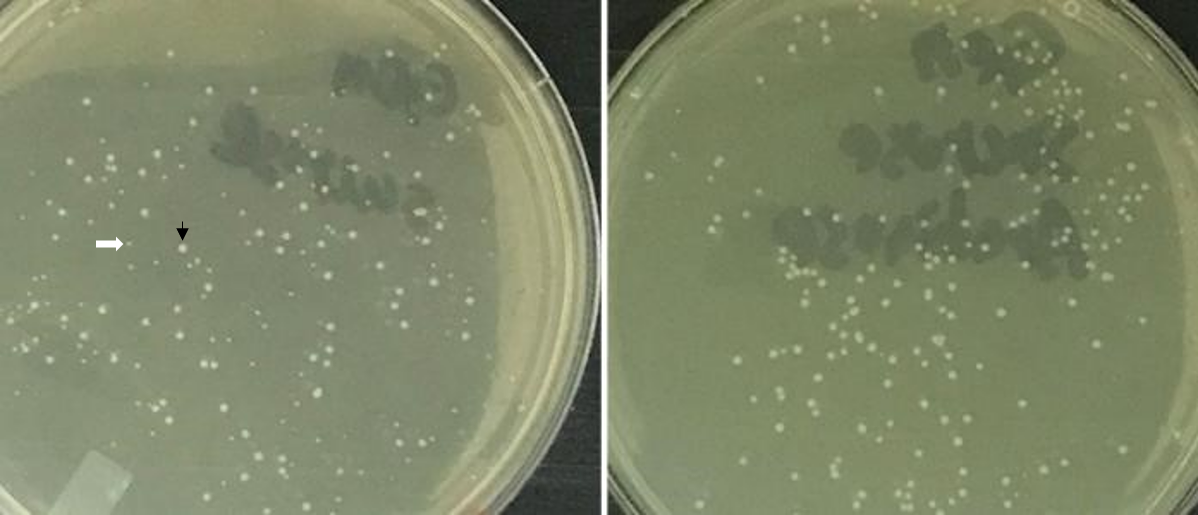
**Supplementary Figure 1.** **Selection of ATCC 17978 pMJG125-*gigAB*** Δ***gigAB* colonies.** pMJG42 Δ*gigAB* was transformed into ATCC 17978 pMJG125-*gigAB*. Left: Colonies were grown on LB plates with sucrose and gentamicin. The larger colonies (black arrow) were found to be ATCC 17978 pMJG125-*gigAB,* and the smaller colonies (white arrow) were found to be ATCC 17978 *ΔgigAB* pMJG125-*gigAB* by sequencing. Right: Colonies were grown on LB plates with sucrose, gentamicin, and 1% arabinose. The clones are ATCC17978 pMJG125-*gigAB ΔgigAB* confirmed by sequencing. The efficiency of *gigA/gigB* deletion is 54.2% for the 1% arabinose condition.
